# Expressions of CXCL12/CXCR4 in Oral Premalignant and Malignant Lesions

**DOI:** 10.1155/2012/516395

**Published:** 2012-01-29

**Authors:** Juan Xia, Na Chen, Yun Hong, Xiaobing Chen, Xiaoan Tao, Bin Cheng, Yulei Huang

**Affiliations:** Department of Oral Medicine, Guanghua School of Stomatology and Institute of Stomatological Research, Sun Yat-Sen University, No. 56, Lingyuanxi Road, Guangdong, Guangzhou 510055, China

## Abstract

*Objective*. The chemokine receptor CXCR4 and its ligand CXCL12 have been suggested to play important roles in the initiation or progression of cancers. The goal of the present study was to investigate alterations of CXCL12/CXCR4 in oral premalignant lesions and oral squamous cell carcinoma (OSCC). *Methods*. In 13 normal oral epithelia, 24 dysplastic oral leukoplakia (OLK), and 40 OSCC specimens, expressions of CXCL12 and CXCR4 were evaluated by immunohistochemistry. *Results*. CXCR4 was expressed in 37.5% of OLK and 60% of OSCC. CXCL12 was detected in 50% of OLK and 62.5% of OSCC. In OLK, CXCR4 positive ratio showed no significant difference from normal epithelia, but the CXCL12 positive ratio was significantly higher. Significant relationship between CXCL12 and CXCR4 was found both in OLK and OSCC. *Conclusion*. Our results indicated that CXCL12/CXCR4 axis may play roles from early steps of oral malignant transformation and contribute to the progress of oral carcinogenesis.

## 1. Introduction

Oral squamous cell carcinoma (OSCC) accounts for approximately 90% of oral malignancies. Approximately 274,000 new cases annually are diagnosed worldwide with only a 50% survival rate over 5 years despite improved methods of diagnosis and therapy [1]. OSCC is of epithelial origin and mostly experiences a premalignant stage. Prevention of this malignancy requires a better understanding of its carcinogenesis process [2, 3]. However, the underlying mechanism still remains enigmatic.

Chemokines, a group of small (approximately 8–17 kDa) chemotactic cytokines that bind to specific G-protein-coupled seven-span transmembrane receptors, are essential mediators of leukocyte migration in the immune system. Chemokines are involved in cellular interactions and tropism in situations frequently associated with inflammation. Chemokines and their receptors play important roles in inflammation, infection, tissue injury, allergy, and cardiovascular diseases. Recently, the importance of chemokines and chemokine receptors has been highlighted in the initiation and progression of cancers [4, 5]. Among those chemokines and their receptors, the stromal cell-derived factor-1 (also CXCL12) and its CX chemokine receptor 4 (CXCR4), a protein frequently overexpressed on the surface of human tumor cells of epithelial origin, have received considerable attention. To date, CXCR4 has been demonstrated to be overexpressed in more than 23 human cancers, including breast cancer, ovarian cancer, and oral cancer. Although CXCR4 is expressed in a broad array of tissues, CXCR4 expression is low or absent in many normal tissues, such as breast and ovary [6, 7]. Significant correlation between CXCR4 expression and lymph-node metastasis was found in OSCC patients. Treatment with CXCL12 enhanced the motility and invasiveness of OSCC cells expressing CXCR4 [8]. Although recently attentions have been paid to the relationship between CXCR4 and oral cancer, until now, no report focuses on the alterations of CXCL12/CXCR4 axis in premalignant stage of oral cancer.

Oral leukoplakia (OLK) is the most common oral precancerous lesion. Leukoplakic oral epithelia provide a useful model for exploring the molecular mechanisms of oral carcinogenesis [3, 9]. In this study, we set out to evaluate the protein expressions of CXCL12 and CXCR4 by immunohistochemistry in surgically resected normal oral epithelia, OLK specimens with epithelial dysplasia, and OSCC tissues. Then, the relationship of CXCL12 and CXCR4 in oral premalignant and malignant lesions was investigated.

## 2. Materials and Methods

### 2.1. Subjects and Samples

A total of 77 subjects, including 24 OLK patients, 40 OSCC patients, and 13 healthy persons, were enrolled in the present study from Guanghua School of Stomatology, Sun Yat-Sen University, China. The subjects were aged between 24 and 78 years. No subjects had radiotherapy, chemotherapy, or other interventional palliative or therapeutic measures prior to sampling. All subjects participated in this study with written consents, and the project was approved by the Institutional Review Board.

All the surgically resected specimens were formalin fixed and paraffin-embedded using conventional histopathological techniques for both histopathological examination and subsequent immunohistochemistry analysis. Histopathological evaluation was performed according to the WHO criteria for histological typing of cancer and precancer of the oral mucosa by the Department of Pathology, Guanghua School of Stomatology.

### 2.2. Immunohistochemistry

The polyclonal primary rabbit antibodies against CXCL12 (1 : 400, Abcam Inc., Cambridge, MA, UK) and polyclonal primary goat antibodies against CXCR4 were used (1 : 300, Abcam Inc., Cambridge, MA, UK). Immunohistochemistry was performed using the SuperPicture, a 3rd Gen IHC Detection Kit (invitrogen, US), and Polink-2 plus Polymer HRP Detection System For Goat Primary Antibody (Golden Bridge International Inc., US) according to the manufacturer's recommendations.

4-*μ*m sections of paraffin-embedded specimens were deparaffinized in xylene (twice) and treated with a graded series of alcohol (100%, 95%, and 80% ethanol/double-distilled H_2_O_2_ (v/v)), and rehydrated in phosphate buffered saline (PBS, pH 7.4). Antigen retrieval was performed by incubating tissue sections immersed in 0.01 M sodium citrate buffer (pH 6.0) in a microwave oven. Endogenous peroxidase was inactivated using 3% hydrogen peroxide for 10 min. After rinsing with PBS, normal goat serum for 10 min was applied to reduce nonspecific staining. The sections were incubated with polyclonal primary antibodies at 37°C for 40 min (CXCL12) or at 4°C overnight (CXCR4). After washing with PBS for 5 min, the sections were incubated for 10 min with biotinylated secondary antibody, washed with PBS, then incubated for 10 min with the streptavidin-horseradish peroxidase (HRP) solution, which binds to the biotin-labelled secondary antibody present to the tissue. After PBS rinsing, staining of sections was performed by incubation for 5 min with 3,3-diaminobenzidine tetrahydrochloride (DAB) in H_2_O_2_. After washing with water, the sections were counterstained with hematoxylin for 3 min. Negative controls for each biopsy were processed in the same manner, using PBS instead of the primary antibody. Human spleen tissue and hepatocarcinoma tissue were used as positive controls for CXCR4 and CXCL12, respectively.

### 2.3. Immunohistochemistry Analysis

Expressions of CXCR4 and CXCL12 were quantified using a visual grading system based on the extent of staining (percentage of positive cells graded on scale from 0 to 4: 0, <5%; 1, 5–25%; 2, 25–50%; 3, 50–75%; 4, >75%) and the intensity of staining (graded on a scale of 0–3: 0, none; 1, weak staining; 2, moderate staining; 3, strong staining). Five representative fields at ×400 magnification were evaluated. A weighted score was assigned to each case by multiplying the score for the percentage of positive cells by the staining intensity score. Cases with a weighted score of less than 1 were considered negative; otherwise they were considered positive [10].

### 2.4. Statistical Analysis

Chi-square analysis was used to estimate statistical difference of positive ratios among different groups during oral carcinogenesis. Probability value of *P* < 0.05 (two-sided) was accepted as statistically significant for all statistical tests carried out using SPSS version 13.0 software (SPSS Inc., Chicago, IL, USA).

## 3. Results

### 3.1. Expressions of CXCR4 and CXCL12 Proteins in OLK and OSCC Tissues

**Figure 1:**
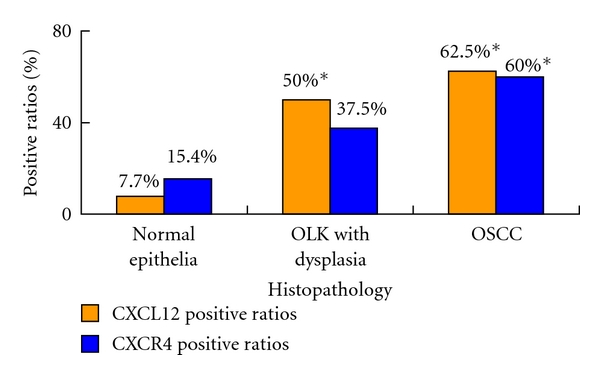
Positive ratios of CXCR4 and CXCL12 in oral premalignant lesions and oral cancers by immunohistochemistry analysis. *P < 0.05, compared with normal epithelia.

**Figure 2:**
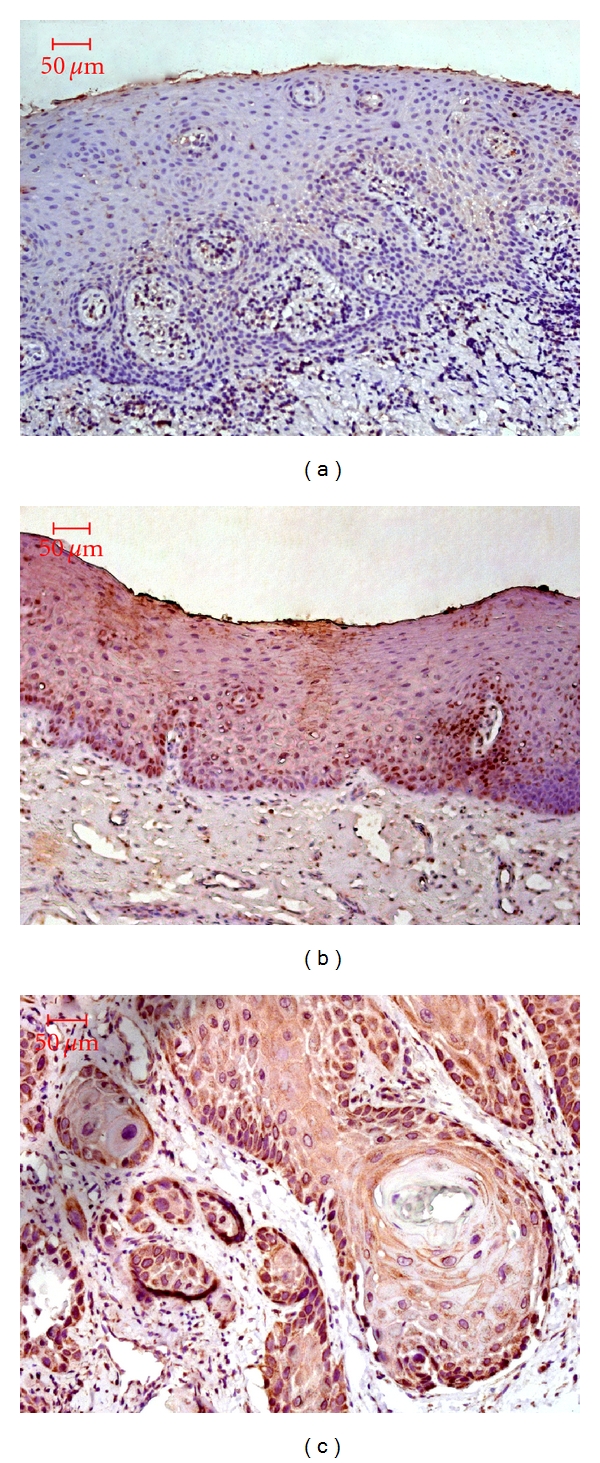
CXCR4 expression in human normal epithelia (a), OLK with dysplasia (b), and OSCC epithelia (c) by method of immunohistochemistry. Original magnifications, ×200.

CXCR4 was expressed in 9 out of 24 OLK cases (37.5%; Figure 1). Of the 40 OSCC cases, 24 (60%) had uniformly detectable CXCR4 immunostaining. However, the positive ratio of CXCR4 was only 15.4% (2 of 13 cases with weak staining) in normal tissue. No significant difference of CXCR4 positive ratio was found between normal tissue and OLK, but in OSCC the positive ratio increased and was significantly higher than that in normal epithelia (*P* = 0.005 < 0.05), without difference from OLK tissues (*P* = 0.081 > 0.05). CXCR4 staining showed a predominantly cytoplasmic distribution and some was also detected in the cellular nucleus (Figure 2). Most normal tissues displayed little or undetectable CXCR4 expression. In both dysplastic and cancerous oral epithelia, the CXCR4 staining was distinct and spread in all the cell layers. Furthermore, mild-moderate staining could also been observed in some vascular endothelial cells within lamina propria and submucosa of dysplastic and cancerous oral epithelia. Figure 2 displayed representative CXCR4 immunostaining with different histopathological grading.

**Figure 3:**
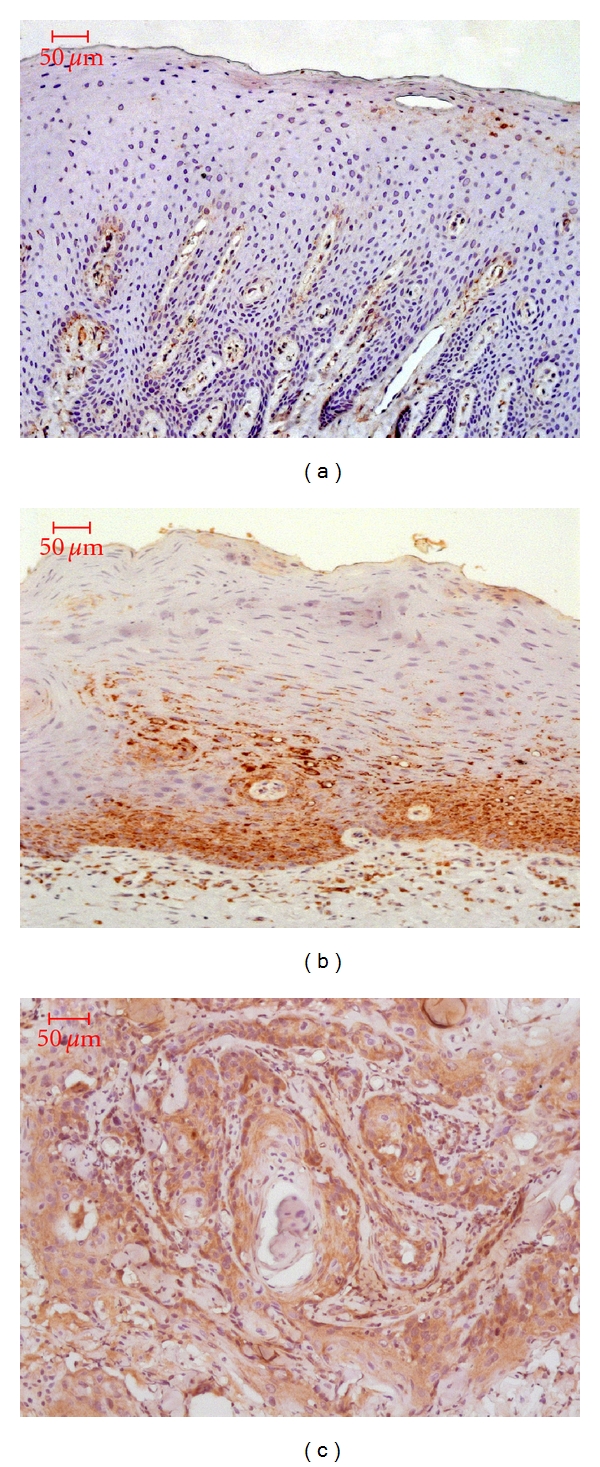
CXCL12 immunostaining in human normal epithelia (a), OLK with dysplasia (b), and OSCC epithelia (c). Original magnifications, ×200.

 CXCL12 staining mainly distributed in the cytoplasm and intercellular substances. Expression of CXCL12 was observed in 12 of 24 OLK (50%; *P* = 0.01 < 0.05) cases and 25 of 40 OSCC cases (62.5%, *P* = 0.001 < 0.05), with significant difference from that in normal epithelia (1 of 13 cases, 7.7%; Figure 1), respectively. No significant difference was indicated between OLK and OSCC tissues (*P* = 0.51 > 0.05).  Figure 3 displayed representative CXCL12 immunostaining in patients' samples.

### 3.2. Correlation of CXCL12 and CXCR4 Expression in OLK and OSCC Tissues

Data analysis found that in 8 OLK and 20 OSCC specimens, which displayed positive staining of CXCR4, expression of CXCL12 could also been detected (Table 1). Significant relationship between CXCL12 expression and CXCR4 expression was found both in OLK (*P* = 0.003 < 0.05) and OSCC specimens (*P* = 0.001 < 0.05).

## 4. Discussion

Oral carcinogenesis is a multistep process and the underlying mechanism is still unclear. Recently, studies revealed that cancer epithelial cells were producing higher levels of a number of chemokines compared with normal epithelial cells and were also expressing high levels of a series of chemokine receptors, to establish a tumor-promoting microenvironment, facilitating tumor-associated angiogenesis and metastasis [11]. Our previous study indicated that CXCR7 expression might be involved in oral carcinogenesis [3]. In this study, we demonstrated for the first time changes of CXCL12/CXCR4 chemokine axis in oral premalignant lesions and their potential roles in oral carcinogenesis.

CXCR4 was expressed in 60% of 40 OSCC cases in our study. Our data were similar to that of two previous reports, which showed that the positive staining ratios were 62.6% and 57.3% in OSCC patients, respectively [12, 13]. All these suggested the tight relationship between CXCR4 and OSCC. CXCR4 expression is upregulated in malignant cells via several mechanisms. Ishikawa et al. [14] found a significant correlation between the expression of CXCR4 and HIF-1 alpha in OSCC tissues. HIF-1 is a heterodimeric transcription factor responsive to oxygen concentrations in tissues and has been shown to upregulate CXCR4 expression. Our results also showed that expression of its ligand CXCL12 was observed in 62.5% of 40 OSCC cases. In 24 CXCR4-positive cases of OSCC, CXCL12, and CXCR4 were coexpressed in 20 cases. When compared with normal tissue, both CXCR4 and CXCL12 positive ratios in OSCC were significantly higher and a significant correlation was found between them. All those indicated the roles of CXCL12/CXCR4 axis in OSCC.

Furthermore, of the 24 oral premalignant lesions (OLK cases), data analysis found that 37.5% displayed CXCR4 positive staining and 50% displayed CXCL12 positive staining. Although CXCR4 positive ratio showed no significant difference between OLK and normal epithelia, the positive ratio of CXCL12 increased significantly from normal epithelia. In 9 CXCR4 positive cases of OLK, 8 cases displayed CXCL12 positive staining. Further data analysis still found a significant relationship between CXCL12 expression and CXCR4 expression in OLK, just similar to that in OSCC tissues. All those demonstrated that CXCL12/CXCR4 axis might begin playing roles from early steps of oral malignant transformation and contribute to the progress of oral carcinogenesis. Binding of CXCL12 to CXCR4 could trigger activation of many downstream targets, including ERK1/2, MAPK, JNK, and AKT effectors. CXCL12/CXCR4 axis activated extracellular signal-regulated kinase (ERK)1/2 and Akt/protein kinase B (PKB) in OSCC cells, and their synthetic inhibitors attenuated the chemotaxis by SDF-1. SDF-1 also activated Src family kinases (SFKs), and its inhibitor PP1 diminished the SDF-1-induced chemotaxis and activation of both ERK1/2 and Akt/PKB [15]. Ligand-stimulated chemotaxis is accompanied by cytoskeletal rearrangements, actin polymerization, polarization, pseudopodia formation, and integrin-dependent adhesion to endothelial cells and other biologic substrates [16–18]. Until now, very few reports concerned the biological functions and related signaling pathways of CXCL12/CXCR4 axis in the progression of OSCC. CXCR4-knockdown OSCC cells showed reduced invasiveness and grew significantly slower than the vector-infected control cells [19]. CXCR4 promoted OSCC migration and invasion through inducing expression of MMP-9, 13 via the ERK signaling pathway [20, 21]. All these suggest that the downregulation of CXCR-4 induces antiproliferative and antiinvasive effects in OSCC and that CXCR-4 might be a useful target molecule for the treatment of OSCC. Uchida et al. [22] showed that subcutaneous administration of AMD3100 (CXCR4 inhibitor) significantly inhibited the lymph node metastases of OSCC cells when they were inoculated into the masseter muscle of nude mice.

Taken together, these findings support the hypothesis that CXCL12/CXCR4 may play important roles in oral premalignant stages, contributing to the progress of oral carcinogenesis. However, much about the mechanisms of CXCL12/CXCR4 axis in oral carcinogenesis still let to be known. Notably, blockage of CXCR4 as well as its ligands CXCL12 during oral cancer promotion could be employed as a new therapeutic strategy in future.

## Figures and Tables

**Table 1:** Association of CXCL12 and CXCR4 expressions in OLK and OSCC tissues.

Histopathology	Total cases (%)	CXCL12-positive cases (%)	CXCL12-negative cases (%)	P*
OLK	24	12 (50%)	12 (50%)	0.003
CXCR4-positive cases (%)	9 (37.5%)	8 (33.3%)	1 (4.2%)	
CXCR4-negative cases (%)	15 (62.5%)	4 (16.7%)	11 (45.8%)	
OSCC	40	25 (62.5%)	15 (37.5%)	0.001
CXCR4-positive cases (%)	24 (60.0%)	20 (50.0%)	4 (10.0%)	
CXCR4-negative cases (%)	16 (40.0%)	5 (12.5%)	11 (27.5%)	

*Chi-square test, relationship between CXCL12 and CXCR4 expressions.
